# Laryngeal tube suction for airway management during in-hospital emergencies

**DOI:** 10.6061/clinics/2017(07)06

**Published:** 2017-07

**Authors:** Haitham Mutlak, Christian Friedrich Weber, Dirk Meininger, Colleen Cuca, Kai Zacharowski, Christian Byhahn, Richard Schalk

**Affiliations:** I Department of Anesthesiology, Intensive Care Medicine and Pain Therapy, Goethe-University Hospital, Theodor-Stern Kai 7-10, 60590 Frankfurt, Germany; II Department of Anesthesiology and Intensive Care Medicine, Medical Campus, University of Oldenburg, Evangelisches Krankenhaus, Steinweg 13-17, 26122 Oldenburg, Germany; III Department of Anesthesiology, Main-Kinzig-Kliniken, Herzbachweg 14, 63571 Gelnhausen, Germany

**Keywords:** Difficult Airway Management, Laryngeal Tube, Supraglottic Airway Devices, In-Hospital Emergencies

## Abstract

**OBJECTIVE:**

The role of supraglottic airway devices in emergency airway management is highlighted in international airway management guidelines. We evaluated the application of the new generation laryngeal tube suction (LTS-II/LTS-D) in the management of in-hospital unexpected difficult airway and cardiopulmonary resuscitation.

**METHODS:**

During a seven-year period, patients treated with a laryngeal tube who received routine anesthesia and had an unexpected difficult airway (Cormack Lehane Grade 3-4), who underwent cardiopulmonary resuscitation, or who underwent cardiopulmonary resuscitation outside the operating room and had a difficult airway were evaluated. Successful placement of the LTS II/LTS-D, sufficient ventilation, time to placement, number of placement attempts, stomach content, peripheral oxygen saturation/end-tidal carbon dioxide development (S_p_O_2_/_et_CO_2_) over 5 minutes, subjective overall assessment and complications were recorded.

**RESULTS:**

In total, 106 adult patients were treated using an LTS-II/LTS-D. The main indication for placement was a difficult airway (75%, n=80), followed by cardiopulmonary resuscitation (25%, n=26) or an overlap between both (18%, n=19). In 94% of patients (n=100), users placed the laryngeal tube during the first attempt. In 93% of patients (n=98), the tube was placed within 30 seconds. A significant increase in SpO_2_ from 97% (0-100) to 99% (5-100) was observed in the whole population and in cardiopulmonary resuscitation patients. The average initial _et_CO_2_ of 39.5 mmHg (0-100 mmHg) decreased significantly to an average of 38.4 mmHg (10-62 mmHg) after 5 minutes. A comparison of cardiopulmonary resuscitation patients with non-cardiopulmonary resuscitation patients regarding gastric contents showed no significant difference.

**CONCLUSIONS:**

LTS-D/LTS-II use for in-hospital unexpected difficult airway management provides a secure method for primary airway management until other options such as video laryngoscopy or fiber optic intubation become available.

## INTRODUCTION

Patients with an unexpected or anticipated difficult airway requiring emergency airway management remain a challenge, even for experienced anesthesiologists and emergency physicians. This condition is responsible for major anesthetic complications, which result in significant morbidity and mortality 1. The most important problems reported among anesthesiologists are difficult or delayed intubation, aspiration of gastric contents and failed intubation 2. A substantial number of in-hospital difficult airway situations occur outside the operating room (OR). Anesthesia outside the OR is a high-risk procedure 3. Airway management outside the OR is mainly influenced by environmental, equipment, and assistance issues and is associated with a 10-fold higher risk of a failed intubation 3-5. In these situations, sufficient oxygenation and ventilation receive the highest priority, followed by avoiding aspiration to prevent serious complications.

If intubation fails, further algorithms should be available. In cases of failure to intubate conventionally, the placement of alternative devices, e.g., supraglottic airway devices (SADs), is recommended in many guidelines for difficult airway 6, 7.

SADs play an important role in the management of patients with difficult airways 8-11. They enable ventilation, even in patients with difficult facemask ventilation, and can furthermore be used as a conduit for endotracheal intubation 10. In the preclinical setting, SADs, such as the laryngeal tube (e.g., the laryngeal tube suction II (LTS II) or LTS-D), have been evaluated and serve as a secure alternative method for maintaining oxygenation and preventing aspiration 12. Intratracheal intubation may then be performed in a standardized environment with adequate equipment. This approach is sometimes defined as “dual” airway management 13.

The aim of the investigation was to evaluate the use of a laryngeal tube for in-hospital emergency airway management.

## METHODS

After approval by the Local Ethical Review Board (455/13), data from 106 adult patients treated using routine anesthesia with an unexpected difficult airway or during resuscitation outside the OR between December 2006 and February 2013 were collected. As a part of a quality assurance program, an observational clinical trial was performed over a period of seven years by anesthetists using a standardized questionnaire. The use of the LTS II/LTS-D in difficult airway situations and in-hospital cardiopulmonary resuscitation (CPR) was examined.

The inclusion criteria consisted of a difficult airway (Cormack Lehane Grade 3-4), CPR, and CPR with a difficult airway. Patients <18 years of age were excluded. After an initial failed intubation, the LTS-II/LTS-D was used as a second-line rescue device by the resuscitation team or the responsible anesthetist in the OR. The time of placement of the LTS-II/LTS-D was measured by the anesthetist or nurse specialist as the time the facemask was removed until the first successful ventilatory hub. The LTS-II/LTS-D was inserted by anesthetists and experienced nurse specialists. The number of intubation attempts before using the LTS-II/LTS-D was at the discretion of the responsible resuscitation team member or anesthetist. Placement of the LTS-II/LTS-D was classified as “failed” if the time for insertion was longer than 120 seconds. The cuff pressure in non-fasting patients was adjusted to 60 cmH_2_O to prevent aspiration; in all other patients, the cuff pressure was gradually decreased until reaching the “best cuff pressure” without leakage or a change in tongue color was achieved. Apart from the LTS-II/LTS-D, a videolaryngoscopy system (Karl Storz GmbH&Co.KG, Tuttlingen, Germany) with a Macintosh blade served as a rescue device for securing the airway.

The main goal of the study was the successful placement of the LTS II/LTS-D with sufficient ventilation in terms of tidal volume after placement. The secondary variables included demographic data, time to placement, classified as <30 seconds, 30-60 seconds or >60 seconds, number of placement attempts (1, 2, 3 or >3), gastric content, S_p_O_2_/_et_CO_2_ development over 5 minutes, subjective overall assessment, prior experience using a laryngeal tube and complications related to the placement of the LTS-II/LTS-D, such as leakage, tongue swelling or bleeding.

Analysis of the data confirmed that a Gaussian distribution was only observed for demographic data such as age and height. Demographic data were summarized as means and standard deviations or numbers and percentages. S_p_O_2_/_et_CO_2_-development and gastric content were summarized as medians and ranges. The statistical analysis was performed using software packages (Microsoft Excel 2013, Microsoft Deutschland GmbH, Unterschleißheim, Germany and GraphPad Prism 6, GraphPad Software Inc., San Diego, CA). Data were analyzed using the non-parametric Mann-Whitney test. Statistical significance was assumed at a probability of a type I error of less than 5% (*p*<0.05).

## RESULTS

Within the seven-year study period, data were available for 106 adult patients (78 males, 28 females) who had their airway managed with the LTS-II/LTS-D in the context of an unanticipated difficult airway and/or in-hospital resuscitation. The mean age was 55 years (standard deviation ±16 years), the mean height was 174 cm (standard deviation ±9.1 cm) and the body mass index was 30 (standard deviation ±8.4 kg/m^2^).

The most frequent indication for the use of an LTS II/LTS-D was a difficult airway situation (75%, n=80), followed by use during CPR (25%, n=26) or an overlap between both (18%, n=19). Predictors for a difficult airway (receding chin, maxillary hypoplasia, micrognathia, retrognathia, reduced mouth opening, short neck, reduced cervical mobility, and obesity) were present in 75 patients (70%). While performing direct laryngoscopy on these patients, a corresponding Cormack and Lehane score of CL3 was observed in 69% (n=52), and a score of CL4 was present in 15% (n=11).

In 94% (n=100) of the cases, users were able to place the laryngeal tube during the first attempt; with two attempts, the success rate increased to 99% (n=105). In 93% (n=99) of the cases, the tube could be placed within 30 seconds; 5% (n=5) of the cases required between 30 and 60 seconds. Only one placement (n=1) required more than 60 seconds. In one case, the laryngeal tube could not be placed. In three cases (3%), the laryngeal tube failed to provide sufficient ventilation after the first placement. In eight cases (7.5%), leakage was detected. In CPR and non-CPR patients, sufficient tidal volume within the first five minutes was measured without significant differences between the two groups.

The majority of the participating anesthetists (87%; n=92) had performed laryngeal tube placement >10 times before, 8% (n=9) had used a laryngeal tube 5-10 times previously, and only 5% (n=5) had placed <5 laryngeal tubes prior to our investigation.

**Figure 1:**
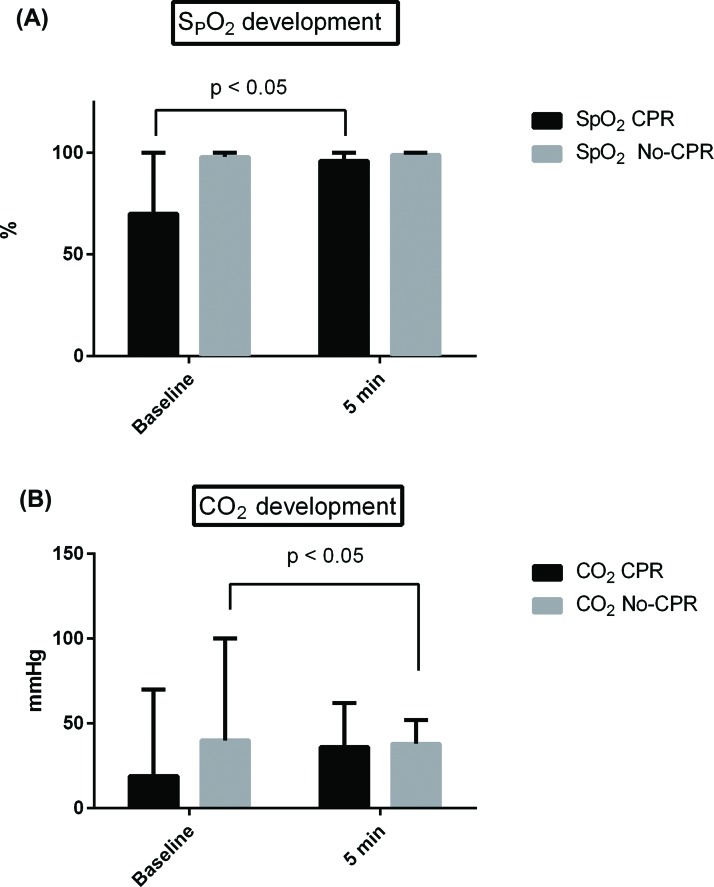
(A) SpO_2_ development and (B) CO_2_ development within the first five minutes in patients with CPR and without CPR.

During the first five minutes, a significant increase in SpO_2_ from 70% (0-100%) to 96% (20-100%) was observed in CPR patients (Figure 1A). An increase in CO_2_ in CPR patients from 19 mmHg (0-70 mmHg) to 36 mmHg (10-62 mmHg) and then a significant decrease in CO_2_ values after five minutes was observed. Patients without CPR exhibited a decrease in CO_2_ values from 40 mmHg (10-100 mmHg) (Figure 1A) to 38 mmHg (30-52 mmHg) (Figure 1B).

An average of 234.2 ml (10-2000 ml) of stomach content was drained through the placed gastric tube, either passively or through active suction, in 28.8% (n=31) of all treated patients. A comparison of CPR patients with non-CPR patients regarding gastric contents showed no significant difference. None of the patients aspirated (if aspiration before LTS placement was suspected, bronchoscopy was performed). For 60% of all placed gastric tubes (n=91), no stomach content was drained.

No complications were recorded.

The laryngeal tube was evaluated by the clinical staff after insertion with a score range from 1 (very bad) to 10 (very good). Placement was evaluated as excellent at 9.7, prevention of leakage was evaluated as 9.6 and ventilation was evaluated as 9.7.

## DISCUSSION

Our data indicate that laryngeal tubes such as the LTS-II/D are a valuable tool in “in-hospital” emergency airway management. Placement is safe and can be easily performed by anesthetists and nurse specialists, even outside the OR, within a short time.

In over 94% of cases, placement was possible during the first attempt and in less than 30 seconds. This finding is inconsistent with recently published data in the preclinical setting. Sunde et al. retrospectively analyzed laryngeal tube insertion in out-of-hospital cardiac arrest and reported 74% of first-attempt placements and overall laryngeal tube insertion of 85%. Nevertheless, only a minority of cases (13%) required more than 30 seconds for placement 14. This difference may be related to the experienced users in our study, the majority of whom had performed more than 10 placements before and used an additional chin lift during insertion of the laryngeal tube. Russo 15 compared three different types of SAD (i-gel, laryngeal mask airway (LMA) Supreme and LTS-D) during elective anesthesia. Although a minimum experience of 15 placements for each device was required, the first attempt success rate was only 53%, and the overall success rate was 70% for the LTS-D; these rates were significantly lower than those found using the i-gel or LMA Supreme. Langenstein and Moeller reported 92% vs 93% success rates for insertion and ventilation using the classic LMA (cLMA) and intubating LMA (ILMA) in patients with a difficult airway 16. Again, this difference may be related to the experienced users in our investigation and the fact that in our institution, the laryngeal tube is the SAD of choice in cases of an unexpected difficult airway in preclinical and clinical settings and is thus implemented in the standard operating procedures for airway management.

No complications associated with the insertion of the LTS were recorded. Our own study group recently published data regarding LTS-associated complications in the preclinical setting 17. Complications consisted of significant tongue swelling as a result of cuff overinflation and gastric overinflation when the laryngeal tube was used without a gastric tube. Our lack of complications may have occurred due to strict cuff pressure monitoring after inflation and also because after securing the airway via the LTS, intubation was performed either via flexible bronchoscopy or (after 2008) video laryngoscope using a technique described earlier 13 under safe environmental circumstances. The fact that video laryngoscopy was implemented in our inner-clinical difficult airway management algorithm after 2009 may explain the small sample size.

The use of a second-generation LTS with a gastric drainage (LTS-II 16 Ch. and LTS-D 18 Ch.) channel may be the reason for the absence of gastric distension. In the OR, when an unexpected difficult airway occurred and SAD utilization was feasible, the surgical procedure was performed with the LTS under intermittent cuff pressure control 18. Furthermore, no aspiration after LTS insertion was recorded, but this possibility cannot be completely ruled out because intubation failure could be consistent with aspiration before placement of the LTS.

The reason for the failed placement in one case remained unclear. Reasons for insufficient ventilation included one case of insufficient depth of anesthesia with bronchospasm and two cases of a reverted tip of the SGA. We observed gastric distension in four patients with multiple intubation attempts and therefore prolonged mask ventilation, but the data were insufficient to correlate these findings. By deepening anesthesia or appropriately placing a gastric tube, ventilation was possible without complications.

Predictors of a difficult airway (receding chin, maxillary hypoplasia, micrognathia, retrognathia, reduced mouth opening, short neck, reduced cervical mobility, and obesity) were present in 70% of cases. This may have occurred because CPR patients were included without a preoperative anesthesiological examination. However, even in the absence of data regarding Mallampati scores or thyromental distance (TMD), our results led to the assumption that these tests have a poor predictive value for a difficult airway in the clinical setting; this finding has been previously described in the literature 19, 20.

We showed that oxygenation and removal of CO_2_ was improved in our population. In CPR patients, the increases in SpO_2_ and CO_2_ are likely related to sufficient CPR by the resuscitation team. In non-CPR patients, the decrease in CO_2_ was significant and may be related to sufficient ventilation via the laryngeal tube. Nevertheless, these significant results are not clinically relevant in our opinion. They only indicate sufficient ventilation. In fact, our data emphasize that the laryngeal tube is a valuable tool for unexpected difficult airway management that can also provide sufficient ventilation outside the OR.

In summary, we highlighted the importance of SGAs in unexpected difficult airway management and in inner-clinical emergency situations, as noted in different difficult airway guidelines 7. We showed that the use of the LTS-D/LTS-II in inner-clinical unexpected difficult airway management provides a secure method to initially secure the airway until other options for securing the airway, such as video laryngoscopy or fiber optic intubation, are available.

### Conflicts of Interest

R.S. and C.B. receive material support for research from VBM Medizintechnik GmbH and Karl Storz GmbH & Co KG, the manufacturers of the laryngeal tube and the C-MAC video laryngoscope and Bonfils intubation fiberscope, respectively. C.B. is a member of the Karl Storz advisory board. None of the other authors has any conflict of interest with products and/or companies mentioned in the manuscript.

## AUTHOR CONTRIBUTIONS

Mutlak H, Byhahn C, Meininger D, Zacharowski K, Weber CF and Schalk R contributed to the writing and preparation of the manuscript. Mutlak H, Meininger D, Schalk R and Byhahn C conceived the study and performed the statistical analysis. Cuca C edited the manuscript as a native speaker.
